# Ensuring vaccine cold chain integrity: A rapid and low-cost test for identifying heat-exposed sucrose-containing vaccines

**DOI:** 10.1016/j.ijpx.2025.100467

**Published:** 2025-12-11

**Authors:** Benediktus Yohan Arman, Andrea Magri, Matteo N. Barbaglia, Lawrence Petherbridge, Jennifer Brook, Tehmina Bharucha, Isabelle Legge, John Walsby-Tickle, Michael Deats, Sneha Banerjee, Sara Mosca, Rajender Jena, Dnyanesh S. Ranade, Shrikrishna R. Chunekar, Kundan D. Patil, Sunil Gairola, Hamid A. Merchant, Robert Stokes, Rutendo Kuwana, Alexandrine Maes, Tim James, Catherine Green, James McCullagh, Pavel Matousek, Céline Caillet, Paul N. Newton, Nicole Zitzmann, Bevin Gangadharan

**Affiliations:** a Department of Biochemistry, University of Oxford, OX1 3QU Oxford, UK; b Kavli Institute for Nanoscience Discovery, University of Oxford, OX1 3QU Oxford, UK; c Clinical BioManufacturing Facility, Nuffield Department of Medicine, University of Oxford, OX3 7JT Oxford, UK; d Department of Clinical Biochemistry, Oxford University Hospitals NHS Foundation Trust, OX3 9DU Oxford, UK; e Department of Chemistry, University of Oxford, OX1 3TA Oxford, UK; f Medicine Quality Research Group, NDM Centre for Global Health Research, Nuffield Department of Medicine, University of Oxford, Oxford OX3 7LG, UK; g Mahidol-Oxford Tropical Medicine Research Unit, Faculty of Tropical Medicine, Mahidol University, Bangkok 10400, Thailand; h Infectious Diseases Data Observatory, Centre of Tropical Medicine & Global Health, Nuffield Department of Medicine, University of Oxford, Oxford OX3 7LG, UK; i Central Laser Facility, Research Complex at Harwell, STFC Rutherford Appleton Laboratory, UKRI, Harwell Campus, OX11 0QX, UK; j Serum Institute of India Pvt. Ltd., 212/2, Hadapsar, Pune 411028, India; k Department of Bioscience, School of Health, Sport and Bioscience, University of East London, Water Lane, London E15 4LZ, UK; l Agilent Technologies LDA UK, Becquerel Avenue, Didcot OX11 0RA, UK; m Regulation and Safety Unit, Regulation and Prequalification Department, Access to Medicines and Health Products Division, World Health Organization (WHO), Geneva, Switzerland

**Keywords:** Vaccine, Potency, Substandard, Sucrose, Glucose, Cold chain, Supply chain, Degradation

## Abstract

Maintaining cold-chain integrity is vital for vaccines to ensure that they remain within the recommended temperature limits during routine storage and transportation. This ensures vaccine stability, efficacy, and avoids degradation. Here, we propose rapid and low-cost tests based on simple glucose assays to detect heat-exposed degraded sucrose-containing vaccines through sucrose's inherent gradual conversion to glucose when exposed to elevated temperatures. Bioluminescent and colorimetric assays and a clinical biochemical analyser for urine samples could successfully determine effects of heat exposure on vaccines by detecting a significant increase in glucose levels. We show that this increase in glucose also correlates with the loss of vaccine potency. When vaccines were incubated at 37 and 45 °C, the bioluminescent assay was able to detect an increase in glucose levels from 12 h of heat exposure. The biochemical analyser could successfully detect increased glucose levels in a COVID-19 vaccine which had been exposed to 37 and 45 °C. Most importantly, the colorimetric assay has the advantage of producing a colour change visually upon simply mixing the vaccine with a reagent without the need for a plate reader or any other sophisticated devices. To our knowledge, this is the first simple, rapid and device-free test of its kind to detect heat-exposed substandard vaccines, making it a potential test for deploying at key points in the supply chain in warm and hot countries to check the integrity of vaccine cold-chain. Although this test does not replace the more definitive lot release assays such as potency assays, it could initially be used as a rapid and low-cost test to identify substandard sucrose-containing vaccines within supply chains, in support of WHO's Prevent, Detect, and Respond strategy.

## Graphical abstract

**Figure:**
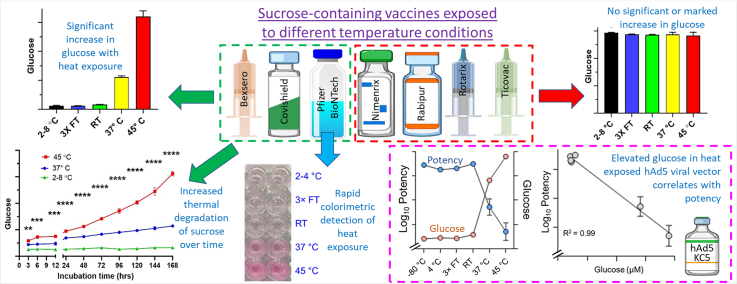

## 1 Introduction

The surge of substandard/falsified (SF) medicines and vaccines coupled with the lack of accessible and affordable methods for their screening and detection in supply chains is a significant problem (Medicine Quality Research Group, University of Oxford, 2022a). One in 10 medical products in low- and middle-income countries are reported to be either substandard or falsified (World Health Organization, 2024). Substandard products are genuine products that fail to meet either quality standards or specifications, or both. In contrast, falsified products are where the products' identity, composition or source is deliberately and fraudulently misrepresented by criminals (World Health Organization, 2024). SF medical products are significant public health security threats and risk higher rates of illness and death, as well as eroding public trust as they are unsafe and ineffective (Newton et al., 2019).

Our Vaccine Identity Evaluation (VIE) Consortium has been evaluating novel techniques for detecting SF vaccines in supply chains (Medicine Quality Research Group, University of Oxford, 2022b). While there are devices for detecting SF tablets, there is a lack of such devices or tests for SF vaccines. We have successfully used spatially-offset Raman spectroscopy (SORS) (Mosca et al., 2023), rapid diagnostic tests (Bharucha et al., 2024), and matrix-assisted laser desorption ionisation time-of-flight mass spectrometry (MALDI-ToF MS) (Arman et al., 2025; Clarke et al., 2024) to differentiate genuine vaccines from falsified vaccine surrogates. Although vaccine manufacturers have in-house reference potency assays, there are no screening devices or tests to detect substandard vaccines due to temperature excursions. The World Health Organization (WHO) estimated that over half of vaccines are wasted globally each year due to temperature control failures, logistics challenges, and shipment-related issues (World Health Organization, 2005) and they recommend vaccine stability testing for extreme environmental conditions including elevated temperature, freeze-thaw and light exposure (World Health Organization, 2006; Dobbelaer et al., 2009). Temperature excursions are the greatest problem since light exposure can be more easily avoided by the secondary and tertiary packaging of vaccine vials and syringes. Cold chain is a crucial aspect of protecting vaccines from deterioration, as improper temperature storage could affect vaccine stability, its efficacy, changes in the safety profile and could also lead to adverse events (Crommelin et al., 2021; Holm and Poland, 2021; Wang et al., 2020). Maintaining the cold chain is expensive and contributes substantially to costs with the pharmaceutical cold chain logistics market size of US $18.61 billion in 2024 and expected to reach $27.11 billion by 2033 (DataM Intelligence, 2025; Wolfson et al., 2008). Successful strategies are needed to generate stable and efficacious dosage forms; these include efforts to stabilise the antigen, selection of adjuvants, and advanced analytical methods to monitor vaccine stability (Kumru et al., 2014).

Vaccines are tested using validated quality control test methods to ascertain their identity, purity, safety and efficacy. Out of the battery of QC methods, potency assay is the crucial lot release parameter to evaluate immunological response which in turn correlates with efficacy of the vaccine. Potency assays for vaccines and medical products rely on biological assays *in vivo* (animal immunogenicity tests) or *in vitro* (cell-based or antibody-binding-based) (Mikołajczyk et al., 2022). Vaccine potency is an indicator of vaccine stability and is a regulatory requirement for product release into the market (Sanyal, 2022). However, these assays are complex, expensive, time-consuming and can be variable with the need for specialist laboratory instruments and trained personnel (Berg et al., 2021; Crommelin et al., 2021; Kis, 2022; Sanyal, 2022; Uddin and Roni, 2021). Vaccine vial monitors (VVM) are thermochromic labels on some vaccine vials which change in colour when exposed to elevated temperatures (Eriksson et al., 2017)(World Health Organization, 2006). VVM are becoming increasingly popular to monitor temperature excursions in supply chains. However, many vaccine syringes and vials do not currently use VVM outside of UNICEF supplied stocks. There are no simple, rapid and low-cost methods to test the vaccine liquid or cake and screen for substandard heat-exposed vaccines in supply chains for most products where VVM are not in-use currently. Such tests, if they reflect potency, would be helpful for health and regulatory authorities as lower cost, faster and simpler alternatives to potency assays until VVM are more widely implemented across all vaccine products globally.

Sugars, such as sucrose and lactose, are common ingredients in approved vaccines where they are used as stabilisers in injectable vaccines (Centers for Disease Control and Prevention, 2021; Food and Drug Administration, 2024) due to their antigen-stabilising and immunogenicity-maintaining properties (Croyle et al., 2001; Gupta et al., 1996; Pelliccia et al., 2016). Sugars are also used in oral vaccines to improve taste. When an aqueous solution of the disaccharide sucrose is heated, it breaks down into its monosaccharides glucose and fructose (Tombari et al., 2007). Drivers of this sucrose hydrolysis include heat and time, both of which are relevant to failure of cold-chain being maintained.

Here we demonstrate three novel, simple, and sensitive methods to detect heat-exposed sucrose-containing vaccines based on the detection of the glucose formed as a product of degraded sucrose molecules: 1) a quantitative bioluminescent glucose assay, 2) a colorimetric glucose assay, which can be used both semi-quantitatively and quantitatively, involving mixing the vaccine with a reagent and then observing a colour change visually, and 3) a quantitative biochemical analyser, which is available in most hospitals worldwide, was used to determine eight analytes, including the levels of glucose in heat-exposed vaccines. Both bacterial and viral vaccines were analysed which were either in liquid or lyophilised forms (Table 1). These quantitative and semi-quantitative glucose assays are not intended to replace the reference vaccine potency assay or VVM and are only presented as surrogate low-cost approaches to empower inspectors to select samples for further investigation.

**Table 1:** Sucrose-containing vaccines used in the study. Data were retrieved from the Electronic Medicine Compendium (EMC), available at https://www.medicines.org.uk/emc and other sources in the public domain.

Vaccine trade name	Description and usage	Manufacturer	Pharmaceutical form	Excipients including sucrose concentration in bold
Rotarix™	Live attenuated rotavirus vaccine	GlaxoSmithKline (GSK)	Oral suspension in squeezable tube, clear and colourless viscous liquid	Sucrose (**715 mg/mL**), disodium Adipate, Dulbecco's Modified Eagle Medium/DMEM (containing phenylalanine, sodium, glucose, and other substances), sterile water
Nimenrix™	Meningococcal groups A, C, W-135 and Y conjugate vaccine	Pfizer Ltd	Powder and solvent for solution for injection in pre-filled syringe	*Powder*: Sucrose (**56 mg/mL** after reconstitution), trometamol *Solvent*: Sodium chloride, water for injection
Rabipur™	Rabies vaccine (inactivated, strain Flury LEP)	Bavarian Nordic A/S	Powder and solvent for solution for injection in pre-filled syringe	*Powder*: Trometamol, sodium chloride, disodium edetate, potassium-L-glutamate, polygeline, sucrose (**60 mg/mL** after reconstitution^a^) *Solvent*: Water for injection
Bexsero™	Meningococcal group-B vaccine (rDNA, component, adsorbed)	GSK	Suspension for injection in pre-filled syringe	Sodium chloride, histidine, sucrose (**20 mg/mL**), water for injections, aluminium hydroxide (hydrated)
TicoVac™ Junior	Tick-Borne Encephalitis Vaccine (whole virus, inactivated)	Pfizer Ltd	Suspension for injection in a pre-filled syringe	Human albumin, sodium chloride, disodium phosphate-dihydrate, potassium dihydrogenphosphate, water for Injection, sucrose (expected to be **≤30 mg/mL** as for TicoVac™ adult dose), aluminium hydroxide, hydrated.
COVISHIELD™	COVID-19 vaccine; Recombinant, replication-deficient chimpanzee adenovirus vector encoding the SARS-CoV-2 Spike (S) glycoprotein	Serum Institute of India	Solution for injection, colourless to slightly brown, clear to slightly opaque and particle-free with a pH of 6.6	L-Histidine, L-Histidine hydrochloride monohydrate, magnesium chloride hexahydrate, polysorbate 80, ethanol, sodium chloride, disodium edetate dihydrate (EDTA), sucrose (**75 mg/mL**)^b^
COMIRNATY™	COVID-19 vaccine; mRNA vaccine (nucleoside modified): 10 micrograms per dose (children 5 to 11 years) and 30 micrograms per dose (ages 12 years and older)	Pfizer Ltd	Concentrate for dispersion for injection	((4-hydroxybutyl)azanediyl)bis(hexane-6,1-diyl)bis(2-hexyldecanoate) (ALC-0315); 2-[(polyethylene glycol)-2000]-N,N-ditetradecylacetamide (ALC-0159); 1,2-Distearoyl-sn-glycero-3-phosphocholine (DSPC); cholesterol, trometamol; trometamol hydrochloride; water for injections; sucrose (**20 mg/mL** (Urru et al., 2025))

a Information obtained from https://www.medsafe.govt.nz/profs/datasheet/r/rabipurinj.pdf.

b Information obtained from https://www.seruminstitute.com/health_faq_covishield.php.

We have recently shown that SORS and MALDI-ToF MS are unable to detect if a sucrose-containing vaccine was exposed to heat (Arman et al., 2025; Mosca et al., 2023). SORS, MALDI-ToF MS and rapid diagnostic tests can aid in detecting falsified vaccines whereas the glucose assays could help in identifying substandard heat-exposed sucrose-containing vaccines, highlighting that these techniques in combination can help to detect SF vaccines.

We conclude that glucose assays could be used as predictors of subpotent heat-exposed vaccines within supply chains in accordance with the WHO's Prevent, Detect, and Respond strategy. Of most importance is the colorimetric assay since it does not require a plate reader or other spectrophotometer, is of very low-cost and therefore could be easily deployed in remote area settings of low- and middle-income countries which may not have access to such instrumentation.

## 2 Materials and methods

### 2.1 Vaccine samples

Five non-COVID-19 vaccines and two COVID-19 vaccines were used in this study (Table 1). Non-COVID vaccines were purchased from the Oxford University Hospitals Pharmacy, Oxford, United Kingdom. All vaccines were in-date and tested before expiry. The COVISHIELD™ (Serum Institute of India) and COMIRNATY™ (Pfizer Ltd) (30 μg for adults and 10 μg for children) COVID-19 vaccines were received from the Serum Institute of India, Pvt. Ltd. and the National Health Service England (NHS England), respectively. Vaccines were stored at the recommended 2–8 °C storage temperature. COMIRNATY™ COVID-19 vaccines were immediately stored in a refrigerator at 2–8 °C when received and used within one month of receipt, according to the manufacturer's recommendations after the frozen vaccine is thawed. Vials and syringes were kept on ice prior to the tests.

A human Adeno5-based viral vector (hAd5 KC5) was generated by single-round infection with a multiplicity of infection of 1 on a hyperflask containing approximately 1 × 10^8^ human embryonic kidney HEK-293 cells. Cells were harvested 48 h post-infection and pelleted at 200 ×*g* for 30 min. Cell pellets were lysed in Cell Lysis Buffer (10 mM Tris, 135 mM sodium chloride, 1 mM magnesium chloride) and freeze/thawed three times. The cell lysate was treated with 250 Units/mL of Benzonase after first thaw and incubated for 30 min at room temperature (RT, recorded as 20 ± 1 °C). The viral vector was then purified by double caesium chloride ultracentrifugation and dialysed three times in Formulation Buffer A438 (10 mM histidine, 7.5 % sucrose, 35 mM sodium chloride, 1 mM magnesium chloride, 0.1 % polysorbate 80, 0.1 mM ethylenediaminetetraacetic acid, 0.5 % (*v*/v) ethanol, pH 6.6). The hAd5 viral vector was stored at −80 °C for long-term storage and was thawed and kept in 4 °C refrigerator just prior to testing.

### 2.2 Experimental settings for vaccine samples

Different techniques were used to measure glucose concentrations in the seven vaccines exposed to different temperature conditions for 7 days and were compared to vaccines stored at 4 °C within the recommended 2–8 °C storage condition which were used as controls. After establishing which vaccines gave rise to measurable glucose levels upon heat treatment after a week, we looked at the sucrose degradation kinetics at 10 different time points over 7 days under the two temperature conditions (37 and 45 °C) that produced significant changes glucose levels in the previous tests, to determine when sucrose degradation could be first detected. For the viral vector sample (hAd5 KC5), we conducted a potency assay in parallel, in order to look at the correlation between glucose levels and potency loss due to heat-exposure.

### 2.3 Vaccine samples exposed to different temperature conditions

One vaccine vial/syringe for each vaccine was exposed to one of the following five temperature conditions in the dark: (i) stored at 4 °C within the recommended 2–8 °C storage condition, (ii) stored at ambient RT for 7 days, (iii) stored in an incubator oven set at 37 °C for 7 days, (iv) stored in an incubator oven set at 45 °C for 7 days, and (v) exposed to three freeze-thaw cycles of 24 h freezing at −70 °C and 1 h thawing at 4 °C per cycle. All samples were immediately stored at 4 °C upon completion of the incubation period. For vaccines which had a separate vial of lyophilised powder and a syringe for the solvent (Nimenrix™ and Rabipur™), both the vial and syringe were exposed to the five conditions prior to mixing/reconstitution, in order to simulate a real-life scenario of temperature excursion on the supply chain. The powdered vaccines were reconstituted with their solvent just prior to analysis.

A degradation test on hAd5 KC5 was performed by incubating glass vials containing 350 μL of the vaccine at RT, 4 °C, 37 °C and 45 °C for 7 days. Vials were also exposed to the same freeze-thaw cycles as described earlier or stored at −80 °C (as a reference control). Each condition was tested using both the colorimetric and potency assays in triplicate.

### 2.4 Sucrose in water exposed to an elevated temperature

Sucrose was made up in ultrapure water from a Milli-Q water purification system (Merck, Germany) at the following concentrations: 20, 87, 200, 350, 540 and 715 mg/mL. An aliquot of each sucrose sample was stored at 4 °C while another aliquot was incubated at 45 °C. These aliquots were 5 mL in volume and stored in the dark at these temperatures for 7 days. All samples were immediately stored at 4 °C post-incubation. Glucose levels were measured in each sample in duplicate using the bioluminescent glucose assay.

### 2.5 Time course for sucrose degradation after heat exposure

Bexsero™, COVISHIELD™, and COMIRNATY™ were aliquoted (120 μL) into 20 microcentrifuge tubes and were separated into two temperature groups (37 and 45 °C). The tubes in each group were exposed to 37 and 45 °C for 3, 6, 12, 24 (1 day), 48 (2 days), 72 (3 days), 96 (4 days), 120 (5 days), 144 (6 days), and 168 (7 days) hours. After completing each incubation time, the tubes were stored at 4 °C. Aliquots of the vaccines were also prepared in the same manner and stored at 4 °C as the 2–8 °C as controls. Following the incubation period, 50 μL of the vaccine samples from each aliquot was assayed, in duplicate, for its glucose concentration.

### 2.6 Bioluminescent glucose assay

The concentration of glucose was measured using the Glucose-Glo Assay (Promega, Madison, WI, USA) according to the manufacturer's protocol. In principle, glucose in the vaccine sample was oxidized by glucose dehydrogenase concomitant with the reduction of NAD^+^ to NADH. In the presence of NADH, a reductase enzyme was used to catalyse the reduction of pro-luciferin to luciferin. The intensity of the light generated was proportional to the amount of glucose and was used to determine its concentration in the vaccine sample.

Vaccine samples (50 μL) and 0–50 μM glucose standards (50 μL) were transferred to wells of a white-bottom 96-well plate (Corning, NY, USA). The reagent buffer was included as a negative control (buffer only) for determining the assay background. Glucose detection reagent (50 μL; a mix of luciferin detection solution, reductase, reductase substrate, glucose dehydrogenase, and NAD) was added to the sample and the plate was shaken for 60 s. The plate was then incubated at room temperature for 60 min. Luminescence was read using a plate-reading luminometer (CLARIOstar, BMG Labtech, Germany). The glucose standard curve was generated using a 4-parameter logistic model using the BMG MARS Data Analysis Software (BMG Labtech) and used to calculate the concentration of glucose in the vaccine samples.

### 2.7 Colorimetric glucose assay

The glucose concentrations in COMIRNATY™ vaccine samples were measured using a colorimetric glucose assay kit (Cell Biolabs, San Diego, CA, USA), according to the manufacturer's protocol. A reaction mix consisting of a colorimetric probe, horseradish peroxidase (HRP), and glucose oxidase was prepared in 1× assay buffer. Glucose standards (50 μL) and vaccine samples (50 μL) were mixed with 50 μL of the reaction mix and prepared in triplicate in a 96-well microtiter plate (Corning). The mixture was incubated for 30 min at 37 °C protected from light. Glucose in the vaccine sample was oxidized by glucose oxidase into D-gluconic acid and hydrogen peroxide. The generated hydrogen peroxide was detected with a colorimetric probe in a reaction catalysed by HRP. The plate was analysed visually by eye and a photograph of the plate was taken using a mobile phone camera. The plate was also read at 540 nm with a microplate reader (CLARIOstar, BMG Labtech). The readouts were blanked to the assay buffer without any glucose standard and Milli-Q water was used as a negative control. The known concentrations of glucose were used to generate a standard curve to calculate the amount of glucose in the sample. A 4-parameter logistic model within the BMG MARS Data Analysis Software (BMG Labtech) was used to calculate the concentration of glucose in the vaccine samples.

### 2.8 Potency determination for heat-exposed vaccines

Potency assays were carried out at the Clinical BioManufacturing Facility in Oxford and could only be performed on the hAd5 viral vector manufactured at the facility. Vaccine potency was determined by infectivity assay and expressed as infectious units (IFU) per mL. Briefly, HEK 293 cells were seeded in 96-well plates at 5.8 × 10^4^ cells/well and subsequently infected with serial dilutions of vaccine test samples. At 48 h post-infection, cells were fixed with methanol, blocked with 1 % BSA, and stained with a mouse monoclonal anti-Hexon antibody (Abcam B025/AD51). Following the incubation with the secondary antibody, Rabbit polyclonal anti-mouse IgG - H&L, HRP-conjugated (Abcam ab6728), positive cells were visualised with DAB staining and enumerated using NyOne (Synentec).

### 2.9 Biochemical analyser

The Abbott Architect c16000 analyser (Abbott Laboratories, Maidenhead, UK) was used to analyse the vaccine samples according to our described method (Brook et al., 2025). The heat-exposed Bexsero™, COMIRNATY™, and COVISHIELD™ vaccine samples (after seven days of exposure to degradation conditions) along with their correctly stored controls were measured with eight separate runs on the instrument. The method optimised for urine specimens was used and the following eight analytes were measured: glucose, calcium, chloride, magnesium, phosphate, potassium, protein and sodium.

### 2.10 Statistical analysis

Ordinary one-way ANOVA with Dunnett's tests was used to compare glucose levels between vaccine samples stored at the recommended 2–8 °C and the other temperature-altered conditions. Statistical analysis was performed using GraphPad Prism v.10.1.2 (GraphPad Software, Boston, MA, USA). A *p*-value less than 0.05 was considered statistically significant.

## 3 Results and discussion

### 3.1 Glucose levels in sucrose-containing vaccines after exposure to different temperature conditions

**Fig. 1:**
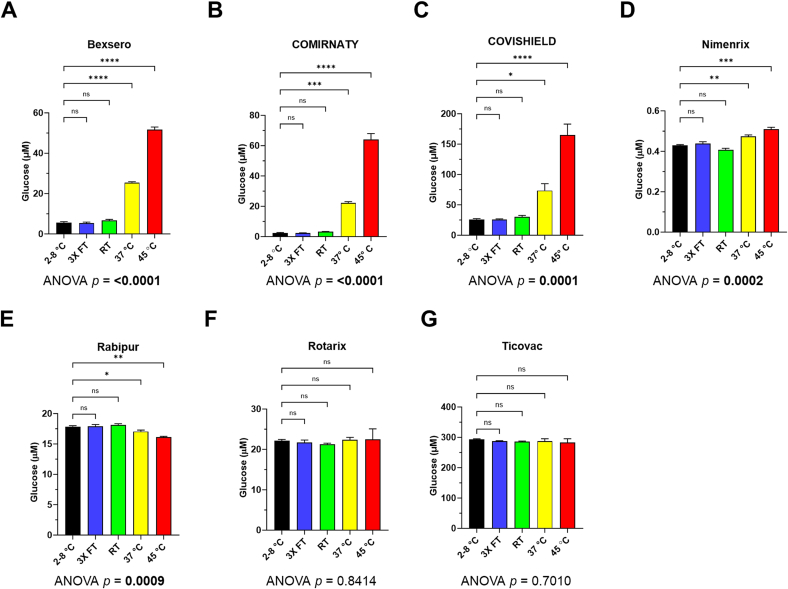
Glucose levels, measured using the bioluminescent assay, of seven sucrose-containing vaccines after exposure to different temperature-altered conditions (coloured bars) compared to the recommended 2–8 °C storage condition (black bar). Error bars show the standard deviations from two measurements for each temperature point. 3× FT, three freeze-thaw cycles; RT, room temperature. Ordinary one-way ANOVA with Dunnett's multiple comparisons tests. ns, not significant; *p < 0.05; **p < 0.01; ***p < 0.005; ****p < 0.001.

Using the bioluminescent assay, glucose concentrations were measured as an indicator of thermal degradation of sucrose after the vaccines were exposed to different temperatures for seven days and compared to vaccines correctly stored at 2–8 °C as recommended by the manufacturers. Among seven vaccines tested, three vaccines (Bexsero™, COMIRNATY™ and COVISHIELD™) showed a clear and statistically significant increase in glucose concentration after exposure to elevated temperatures of 37 and 45 °C (Fig. 1. A-C). A smaller increase was observed for Nimenrix™ but was significant at both 37 and 45 °C (Fig. 1 D). The increase was recorded as 4.5-, 9.6-, 2.8-, and 1.1- fold in Bexsero™, COMIRNATY™, COVISHIELD™, and Nimenrix™, respectively, after seven days of incubation at 37 °C compared to the vaccine samples stored at 2–8 °C. After incubation at 45 °C, a higher increase in glucose was observed compared to incubation at 37 °C, with a 9.2-, 27.6-, 6.3-, and 1.2-fold change in Bexsero™, COMIRNATY™, COVISHIELD™, and Nimenrix™, respectively, compared to the 2–8 °C storage condition (Table S1). However, no significant increase in glucose after heat exposure was observed for Rabipur™, Rotarix™, and Ticovac™ (Fig. 1 *E*-G). Vaccines which had undergone freeze-thaw were also investigated since this environmental condition is recommended for vaccine stability testing (World Health Organization, 2006; Dobbelaer et al., 2009). Exposure to three freeze-thaw cycles and room temperature conditions after seven days did not result in significant changes in glucose levels in any of the vaccines tested (Fig. 1).

A significant increase in glucose after seven days of exposure to elevated temperatures of 37 and 45 °C was observed for COMIRNATY™, COVISHIELD™ and Nimenrix™. For Nimenrix™, although the increase was significant, the increase in glucose was only 1.1- and 1.2-fold for 37 and 45 °C whereas for the other vaccines the fold change was considerably higher (2.8- to 9.6-fold for 37 °C and 6.3- to 27.6 fold for 45 °C; Table S1). For Rabipur™, another lyophilised vaccine, no significant increase in glucose was observed. Lyophilised vaccines have been shown to have good stability at 37 °C for up to one month (Berg et al., 2021). The lower increase in glucose for Nimenrix™ and no increase in glucose for Rabipur™ is likely to be due to these vaccines being lyophilised; water is needed for sucrose hydrolysis and with such a small fold change for Nimenrix™ it may be challenging to use glucose assays to detect its exposure to heat.

No significant increase in glucose was observed also for Rotarix™ and Ticovac™ (Fig. 1) suggesting that sucrose degradation depends on vaccine formulation (Berg et al., 2021). Rotarix™ is a highly viscous liquid including a thickening agent disodium adipate (Kumar et al., 2021). Unlike all the other injectable vaccines tested, Rotarix™ is given orally and contains sucrose to improve taste. The high viscosity of the vaccine and the extremely high level of sucrose compared to injectable vaccines (Table 1) could be the reason why no significant increase in glucose was observed. Ticovac™ contains human serum albumin that functions as sheer protection and thermal stability enhancer (Orellana, 2002). The presence of this excipient may have helped to reduce the thermal degradation of sucrose.

No significant change in glucose levels was observed for all vaccines when exposed to freeze-thaw cycles and stored at ambient room temperature of 20 °C for seven days. However, damage from accidental freezing can result in potency loss for freeze-sensitive vaccines (Matthias et al., 2007) and this could not be detected using the glucose assays. A drop in infectivity has been shown for a ChAdOx1 vaccine exposed to 22 °C from 35 days onwards and for a ChAdOx2 vaccine from 180 days onwards (Berg et al., 2021). These vaccines had the same excipients as COVISHIELD™ and the same sucrose concentration of 75 mg/mL. For all vaccines tested in our study, we did not see any change in glucose after 7 days at RT (∼20 °C). However, we have not tested if glucose increases after 35 days at this ambient temperature since it is unlikely that vaccines would be left for so long at RT in the supply chain.

No significant increases in glucose concentrations were observed for Rabipur™, Rotarix™ and TicoVac™ (Fig. 1) and this is likely due to these vaccines being in powdered form, highly viscous and having albumin as a thermal stability enhancer, respectively. The powdered vaccines (Rabipur™ and Nimenrix™), were not made up in the solution for injection. In a real-life scenario, powdered vaccines would only be reconstituted by the health professional prior to administration and would never be reconstituted in the supply chain. Therefore, these powdered vaccines were exposed to heat prior to reconstitution to replicate what could happen in the supply chain. Sucrose hydrolysis requires water which explains why there was no marked increase in glucose concentration for both powdered Rabipur™ and Nimenrix™. However, water is present in both Rotarix™ and TicoVac™ and therefore excipients in these vaccines are likely to be protecting sucrose from heat-induced hydrolysis. To confirm that excipients protect sucrose from heat-induced hydrolysis, sucrose was made up at different concentrations in water, free from any excipients, and exposed to an elevated temperature. Levels of glucose were measured in these sucrose samples made up in water when stored at both 4 and 45 °C (Fig. S1). Sucrose solutions were made up at various concentrations from 20 to 715 mg/mL since they cover the sucrose concentrations commonly used in vaccines, *e.g.* Bexsero (20 mg/mL), Spikevax (87 mg/mL), RotaTeq (540 mg/mL) and Rotarix (715 mg/mL). The sucrose solutions stored at 4 °C had very low levels of glucose (below 10 μM). However, for all solutions stored at 45 °C, there was a large increase in glucose of around 100-fold (Fig. S1) unlike the vaccine data with relatively small increases in glucose (Fig. 1). This indicates that all concentrations of sucrose commonly used in vaccines could show an increase in glucose with heat exposure. However, we either observed a relatively lower increase in glucose of 1.2 to 27.6-fold (Table S1, Fig. 1A-D) or no significant increase at all (Fig. 1E-G). The sucrose solutions made up in water did not have any other excipients and therefore the lower or no increase in glucose observed for the vaccines must be due to other excipients which are reducing sucrose hydrolysis (Table 1). Further work is needed to test temperatures below 37 °C to identify the lowest temperature at which glucose rises could be detected.

### 3.2 Analysis of thermal degradation of sucrose over time using the bioluminescent glucose assay

**Fig. 2:**
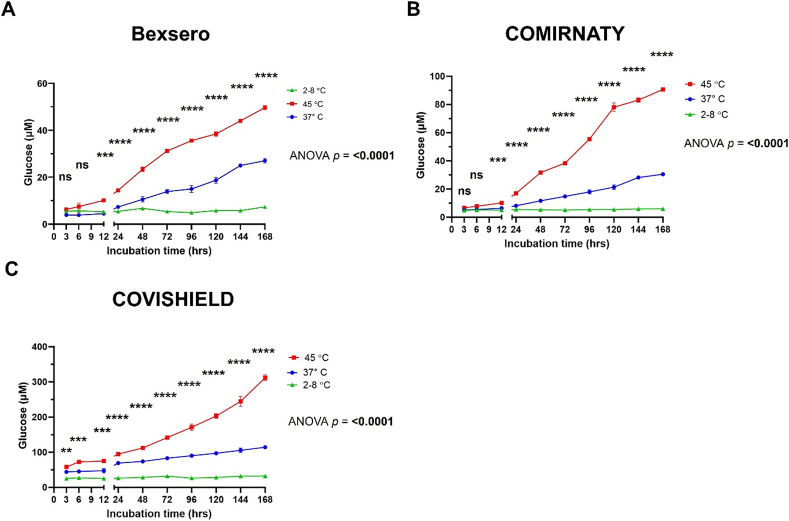
Analysing the thermal degradation of sucrose over 7 days for (A) Bexsero™, (B) COVISHIELD™, and (C) COMIRNATY™ at 37 °C (blue line) and 45 °C (red line), compared to the recommended storage temperature of 2–8 °C (green line) using the bioluminescent glucose assay. Error bars show the standard deviations from two measurements. Ordinary two-way ANOVA with Dunnett's multiple comparison test. ns, not significant; **p < 0.01; ***p < 0.005; ****p < 0.001. (For interpretation of the references to colour in this figure legend, the reader is referred to the web version of this article.)

After establishing which vaccines gave rise to measurable glucose levels upon heat treatment after a week, we proceeded to measure those vaccines (Bexsero™, COMIRNATY™, and COVISHIELD™) over a seven-day time-course to determine when sucrose degradation could be first detected by the bioluminescent glucose assay. This time-course experiment was performed to help to understand the earliest time point at which inspectors could use the glucose assay to determine vaccine heat-exposure. Thermal degradation of sucrose in all three vaccines could be observed by increasing levels of glucose when exposed to 37 and 45 °C. Significant increases in glucose levels were detected after 12 h for Bexsero™ and COMIRNATY™, and 3 h for COVISHIELD™ (Fig. 2). Vaccine samples were also kept at 4 °C to compare with the same time points when the vaccines were stored at elevated temperature (green plot in Fig. 2). These 4 °C samples at the same time points confirm that there was no elevation of glucose over time for sucrose containing vaccines correctly stored at the recommended 2–8 °C.

Samples were exposed to elevated temperature for pre-set times and then stored at 4 °C to replicate what can happen in supply chains. If a vaccine was inappropriately left at ambient temperature, which can be as high as 37 to 45 °C in some countries, it would not be at this elevated temperature during the whole journey on the supply chain and would be returned to 2–8 °C prior to and when arriving at the vaccination centre. In this scenario, although the vaccine is at the correct temperature when received, the end user would be unaware, if the vaccine lacks VVM, of the vaccine's temperature excursion. In our study, by storing the heat-exposed vaccines back to 4 °C, we replicated what does sometimes occur and we show that the glucose assays can successfully detect these substandard vaccines.

Sucrose degradation could be detected as early as 12 h post-incubation when Bexsero™, COVISHIELD™ and COMIRNATY™ vaccines were exposed to 37 and 45 °C (Fig. 2.). In the case of COVISHIELD™, a significant change in glucose (*p* < 0.01) was observed after only 3 h of incubation at these temperatures (Fig. 2C) with glucose concentration changes of 18.5 and 32.4 μM for 37 °C *vs* 4 °C and 45 °C *vs* 4 °C, respectively. The bioluminescent glucose assay was sensitive enough to detect these small changes. The assay is capable of quantifying glucose levels down to 0.0031 μM, which is the lowest standard concentration used in the assay. Although a plate reader was required to read the bioluminescence, the assay is straightforward and many medicine regulators and vaccine manufacturers would have this device since they are already used for vaccine potency assays. While both potency assays and the bioluminescent glucose assay use a plate reader, the glucose assay is lower cost, faster and easier to carry out. Further work is needed to test different temperature and time combinations within the range of temperatures expected in warmer countries.

### 3.3 Colorimetric glucose assay to detect glucose levels in sucrose-containing vaccines

**Fig. 3:**
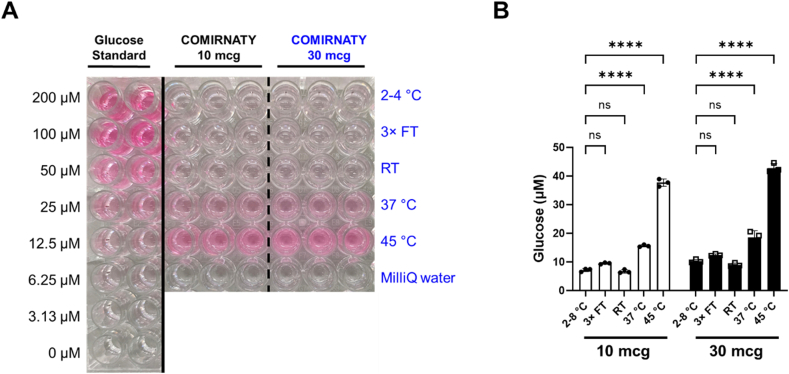
A colorimetric glucose assay was used to measure levels of glucose in 30 μg adult and 10 μg children's COMIRNATY™ vaccines after exposure to different temperature conditions. (A) Photograph of the plate showing an increase in glucose at 37 °C and a further increase at 45 °C. The difference in colour intensity for 37 °C and 45 °C compared to 2–8 °C storage can be observed by eye without the need for a plate reader. (B) The intensity of the colour change was also analysed using a spectrophotometer at 540 nm and the concentrations of glucose in the vaccine samples were determined based on the glucose standards. The levels of glucose in temperature-altered conditions were compared to the recommended 2–8 °C storage temperature. Error bars show the standard deviations from three measurements for each temperature point. 3× FT, three freeze-thaw cycles; RT, room temperature. Ordinary one-way ANOVA with Dunnett's multiple comparisons tests. ns, not significant; ****p < 0.001.

A simpler colorimetric glucose assay was used to determine the glucose levels in COMIRNATY vaccines for both the 30 μg adult and 10 μg children's vaccines (Fig. 3, Table 1). The development of colour intensity along with the increasing level of glucose could be observed visually by eye (Fig. 3A) and could detect vaccine vials exposed to elevated temperatures of 37 and 45 °C (Fig. 3B). A similar increase in glucose was observed in 30 μg adult and 10 μg children's vaccines which both are manufactured with the same amount of sucrose.

To avoid the use of a plate reader and to decrease costs further, we investigated the use of a rapid and simpler colorimetric glucose assay. This assay could also be used with a plate reader but only requires absorbance measurements instead of luminescence and such spectrophotometers are more widely available globally and are of lower cost. Most importantly, the changes in colour intensity seen with the heat-exposed vaccines and comparing this to the pink colour of the glucose standards, it was possible to determine the concentration of glucose semi-quantitatively and visually without the need for any device (Fig. 3A). The method simply involves mixing vaccine with a reagent, incubating at 37 °C for 30 min and any colour change to pink would indicate that the vaccine was exposed to heat. The reagents in this kit could be used along with a reference of colours expected for correctly stored (colourless) and heat-exposed (pink) vaccines. This is the first rapid and device-free assay which correlates with vaccine potency and could significantly aid inspectors in countries where instrumentation is lacking. Although the sensitivity of this assay is lower than the bioluminescent assay, it can successfully determine vaccines exposed to both 37 °C and 45 °C. The colorimetric glucose assay used in this study is distributed worldwide, including to over a dozen LMICs such as Nigeria, India, Pakistan and Egypt, which have average high temperatures exceeding 40 °C during summer months.

Elevated temperatures have been shown to degrade an in-house manufactured mRNA vaccine (Raffaele et al., 2022). A decrease in mRNA integrity and increase in fragmentation were observed after four days of incubation at 37, 45, and 60 °C (Raffaele et al., 2022). At this same 4 days (96 h) timepoint, we were able to successfully detect a significant increase in glucose for the COMIRNATY™ mRNA vaccine at both 37 and 45 °C (Fig. 2B). This suggests that our novel approach using glucose assays could be used to detect heat-exposed mRNA vaccines which have reduced mRNA integrity and increased fragmentation. The stability of mRNA, as of percentage of intact RNA, correlates with *in vitro* potency as both were rapidly decreased when exposed to increasing temperature from 25 to 45 °C (Tong et al., 2024). All mRNA vaccine manufacturers use sucrose and similar excipients (lipids and salts) and therefore the glucose assays are likely to work for all mRNA vaccines.

Our proposed use of the colorimetric glucose assay is the fastest and lowest cost assay for heat exposed vaccines. To our knowledge, this is the first device-free test which can detect heat-exposed vaccines and correlates with vaccine potency. This assay simply involves mixing vaccine with a reagent and visualising any colour change to pink would indicate heat-exposed vaccines. An oven is required to incubate the mixed sample and ovens are present in laboratories of all medicine regulators. However, even an oven may not be needed in countries where the ambient temperature is high enough to show the colour change to pink.

### 3.4 Correlation of potency with glucose for heat-exposed vaccines

**Fig. 4:**
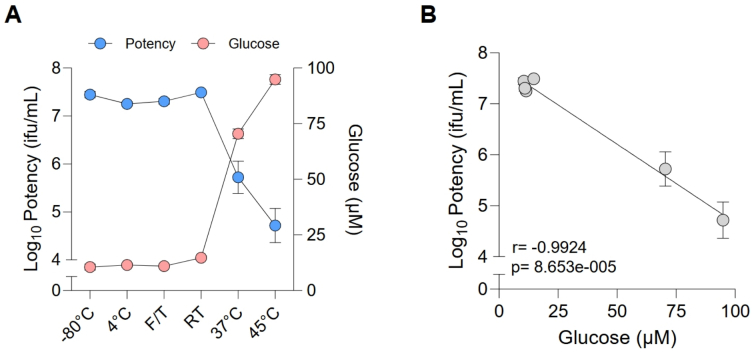
Assessment of the impact of degradation conditions on hAd5 viral vector potency indicated by infectivity and the resultant glucose levels. (A) Correlation between vaccine potency and glucose levels, (B) Pearson correlation between vaccine potency and glucose levels.

To assess whether glucose levels correlate with vaccine potency, a human Adenovirus 5-based viral vector formulated in a buffer containing 7.5 % sucrose was exposed to various temperature-altered conditions. Storage at 4 °C, RT, or three times FT had no impact on viral potency compared to the reference sample stored at −80 °C (Fig. 4A); similarly the detected glucose levels showed no significant deviation compared to the reference sample. Exposure of samples to higher temperatures (37 °C and 45 °C) resulted in reduced potency, with a 2-log decrease for 37 °C and a 3-log reduction for the 45 °C incubation. Glucose levels from those samples were elevated, between 5- and 11-fold (Fig. 4A). Furthermore, the correlation analysis confirmed that degradation-mediated glucose levels correlate significantly with vector infectivity, confirming that glucose is potentially a good predictor for vaccine potency (Fig. 4B).

Previous work assessing adenovirus-vectored vaccines exposed to temperatures ranging from 4 to 45 °C, using an *in vitro* infectivity assay, reported a rapid loss of infectivity at 30 and 45 °C in less than 10 days of exposure. Only a small decrease in infectivity was observed at 22 °C and no loss of infectivity was seen over five freeze-thaw cycles (Berg et al., 2021). Therefore, while the glucose assays were not able to detect vaccines which had been exposed to freeze-thaw cycles, the potency may have been unaffected, as observed in our potency correlation data for the hAd5 viral vector (Fig. 4). A rapid initial loss of infectivity has also been observed for a measles vaccine when exposed to temperatures above ambient (Mann et al., 1983). Furthermore, a case report assessing the stability of an adenovirus-vectored ChAdOx1-S vaccine stored at 21 °C for 18 h before vaccination reported the same safety profile and efficacy as the vaccines stored at the recommended temperature (Mikołajczyk et al., 2022). This maintained efficacy at 21 °C is in line with our observed no significant increase in glucose at 20 °C ambient temperature. We incubated the vaccine at 20 °C for a much longer period of 7 days (Fig. 1). However, for 20 °C exposure over the same 18 h, the increase in glucose is likely to be minimal or not significant based on our data for exposure to 37 °C (at least for Bexsero™ and COMIRNATY™; Fig. 2A and Fig. 2B).

A limitation of this study is that potency assays, and corresponding glucose assays, were only performed with one sample, the hAd5 viral vector, since it was not possible for the Clinical BioManufacturing Facility to carry out potency assays on vaccines not manufactured at the facility.

### 3.5 Glucose assays using a biochemical analyser

Using the biochemical analyser, glucose could be successfully detected in COVISHIELD™ exposed to 37 °C (at the lower limit of quantitation 60 μM) and 45 °C (140 μM). Both of these measured glucose concentrations were similar to the levels detected by the bioluminescent glucose assay (Fig. 1C). Glucose could also be detected at even higher levels in COVISHIELD™ samples exposed to 100 °C (data not shown since vaccines would never be exposed to such a high temperature on the supply chain). However, glucose could not be detected and quantified in Bexsero™ and COMIRNATY™ samples.

A biochemical analyser was repurposed to detect analytes in vaccine excipients. This analyser can measure glucose in clinical samples (along with 7 other analytes), is very low cost (below £10 to measure all 8 analytes) and is widely available in most diagnostic laboratories worldwide. Although the instrument did not have the same level of sensitivity compared to the bioluminescent or colorimetric assays, it has the advantage of confirming many analytes contained in the genuine vaccine and can be used to evaluate vaccine authenticity (Table 2 and Table S2). While the biochemical analyser was unable to detect glucose in heat exposed Bexsero and COMIRNATY™, it was able to successfully detect glucose in heat-exposed COVISHIELD™ (Table 2). This was expected since the bioluminescent assay showed higher levels of glucose after heat exposure for COVISHIELD™ (Fig. 1C), which was within the working range of the biochemical analyser, compared to Bexsero (Fig. 1A) and COMIRNATY™ (Fig. 1B). Also, the concentration of sucrose in COVISHIELD™ is 3.75 times higher than both Bexsero and COMIRNATY™ (Table 1). It is possible that the biochemical analyser may be able to detect glucose in vaccines with a similar sucrose concentration as COVISHIELD™. The manufacturer of this vaccine, Serum Institute of India, has also recently manufactured the R21 malaria vaccine which contains sucrose although its concentration is not in the public domain. It is possible that a biochemical analyser could detect heat exposure for this vaccine, and other sucrose-containing vaccines, assuming that the sucrose concentrations are similar to that of COVISHIELD™.

**Table 2:** Concentrations of glucose measured in vaccine samples using an Abbott Architect c16000 biochemical analyser after exposure to different altered temperature conditions. Values in bold indicate if the analytes could be detected and quantified showing their mean concentrations (± standard deviation) of eight measurements. All other values with the less than symbol (<) were below the limit of quantitation and the lower limit of quantitation is shown.

Vaccine	Glucose concentration (mM)				
	4 °C	3× FT	RT	37 °C	45 °C
Bexsero⁎	< 0.06	< 0.06	< 0.06	< 0.06	< 0.06
COMIRNATY	< 0.06	< 0.06	< 0.06	< 0.06	< 0.06
COVISHIELD	< 0.06	< 0.06	< 0.06	**0.06 ± 0.01**	**0.14 ± 0.01**

⁎ Calculation based on six measurements due to the limited sample; 3× FT, three cycles of freeze-thaw; RT, room temperature (20 ± 1 °C).

The other analytes detected and quantified by the biochemical analyser helped to determine an analyte fingerprint for the vaccines and we have shown how this is a low-cost way to distinguish genuine vaccines from falsified surrogates (Brook et al., 2025). The analytes identified and quantified for the three vaccines act as a fingerprint to confirm authenticity (Supplemental Table 2). All vaccines tested contain sodium chloride and as expected, both sodium and chloride ions were detected. Although Bexsero™ does not contain potassium, a positive result was seen possibly due to interference from other excipients which is not a problem since the result was consistent among all runs. Such false positives may occur since the analyser is intended for urine samples instead of vaccines which have a very different sample matrix. COMIRNATY™ tested positive for protein even though no protein is expected in this vaccine. As we have discussed separately, an explanation for this false positive in mRNA vaccines could be due to benzethonium chloride used for the protein method which interacts with lipids in the vaccine and disrupts the lipid nanoparticles (Brook et al., 2025). Magnesium chloride is present in COVISHIELD™ and as expected magnesium was detected. The Oxford COVID-19 ChAdOx1-S vaccine has the same excipient list as COVISHIELD™ and we have shown that magnesium ions could also be detected in this vaccine (Brook et al., 2025).

Since sucrose is composed of both glucose and fructose, levels of fructose could have also been detected instead of glucose. However, since glucose assays are far more common, more affordable and the biochemical analyser is unable to measure fructose, we decided to only focus on detecting glucose. The approaches described may also be expanded to detect degradation in lactose-containing liquid medicines (Centers for Disease Control and Prevention, 2021), as lactose breaks down into glucose and galactose when heated. Galactose assays are also available but have the same disadvantages as fructose assays.

## 4 Conclusion

We have demonstrated that the surrogate measurement of glucose, as a product of sucrose degradation, could be used as a predictor of sucrose-containing vaccines exposed to elevated temperatures during transportation and storage at various points in supply chains. This novel approach was found to successfully work for most of the vaccines tested. Importantly, we have shown that the increase in glucose correlates significantly with vector infectivity, confirming that this novel marker for heat-exposed sucrose-containing vaccines is a good predictor for loss of vaccine potency due to temperature excursions in the supply chain. We propose that the detection of glucose could potentially be an endogenous indicator of temperature exposure for some sucrose-containing vaccines and thus indicative of the cold chain failure.

The colorimetric glucose assay used in this study is low cost and rapid with results after only 30 min. This is the first test which does not require any sophisticated device, can successfully detect heat-exposed vaccines and correlates with vaccine potency making it ideal in resource-limited settings, such as in LMICs.

Our suggested glucose assays could be used by medicine regulators, vaccine manufacturers, hospitals, and inspectors as an initial screening test to help prevent substandard vaccines from being used.

## CRediT authorship contribution statement

**Benediktus Yohan Arman:** Writing – review & editing, Writing – original draft, Visualization, Validation, Methodology, Investigation, Formal analysis, Data curation. **Andrea Magri:** Writing – review & editing, Visualization, Validation, Resources, Methodology, Investigation, Formal analysis, Data curation. **Matteo N. Barbaglia:** Writing – review & editing, Investigation. **Lawrence Petherbridge:** Writing – review & editing, Investigation. **Jennifer Brook:** Writing – review & editing, Investigation. **Tehmina Bharucha:** Writing – review & editing. **Isabelle Legge:** Writing – review & editing. **John Walsby-Tickle:** Writing – review & editing. **Michael Deats:** Writing – review & editing. **Sneha Banerjee:** Writing – review & editing. **Sara Mosca:** Writing – review & editing. **Rajender Jena:** Writing – review & editing, Resources. **Dnyanesh S. Ranade:** Writing – review & editing, Resources. **Shrikrishna R. Chunekar:** Writing – review & editing, Resources. **Kundan D. Patil:** Writing – review & editing, Resources. **Sunil Gairola:** Writing – review & editing, Resources. **Hamid A. Merchant:** Writing – review & editing. **Robert Stokes:** Writing – review & editing. **Rutendo Kuwana:** Writing – review & editing, Resources. **Alexandrine Maes:** Writing – review & editing, Resources. **Tim James:** Writing – review & editing, Project administration. **Catherine Green:** Writing – review & editing, Resources, Project administration. **James McCullagh:** Writing – review & editing, Project administration, Funding acquisition. **Pavel Matousek:** Writing – review & editing, Project administration, Funding acquisition. **Céline Caillet:** Writing – review & editing, Project administration, Funding acquisition. **Paul N. Newton:** Writing – review & editing, Writing – original draft, Project administration, Funding acquisition. **Nicole Zitzmann:** Writing – review & editing, Writing – original draft, Supervision, Project administration, Funding acquisition. **Bevin Gangadharan:** Writing – review & editing, Writing – original draft, Visualization, Validation, Supervision, Project administration, Methodology, Investigation, Formal analysis, Data curation, Conceptualization.

## Declaration of competing interest

The authors declare the following financial interests/personal relationships which may be considered as potential competing interests:

Isabelle Legge reports financial support was provided by Two anonymous donor families. John Walsby-Tickle reports financial support was provided by Two anonymous donor families. Bevin Gangadharan reports financial support was provided by Two anonymous donor families. Isabelle Legge reports financial support was provided by 10.13039/100001275Oak Foundation. John Walsby-Tickle reports financial support was provided by 10.13039/100001275Oak Foundation. Bevin Gangadharan reports financial support was provided by 10.13039/100001275Oak Foundation. Sneha Banerjee reports financial support was provided by 10.13039/100004423World Health Organization. Sara Mosca reports financial support was provided by World Health Organization. Michael Deats reports financial support was provided by 10.13039/100010269Wellcome Trust. Celine Caillet reports financial support was provided by Wellcome Trust. Paul N. Newton reports financial support was provided by 10.13039/100010269Wellcome Trust. Pavel Matousek reports financial support was provided by 10.13039/100004423World Health Organization. James McCullagh reports financial support was provided by Two anonymous donor families. Paul N. Newton reports financial support was provided by Two anonymous donor families. Nicole Zitzmann reports financial support was provided by Two anonymous donor families. Benediktus Yohan Arman reports financial support was provided by Indonesia Endowment Fund for Education. Bevin Gangadharan reports financial support was provided by Oxford Glycobiology Endowment. Bevin Gangadharan reports financial support was provided by Medical and Life Sciences Translational Fund. James McCullagh reports financial support was provided by 10.13039/100001275Oak Foundation. Paul N. Newton reports financial support was provided by 10.13039/100001275Oak Foundation. Nicole Zitzmann reports financial support was provided by 10.13039/100001275Oak Foundation. Pavel Matousek reports a relationship with Agilent Technologies that includes: consulting or advisory. Robert Stokes reports a relationship with Agilent Technologies that includes: employment. If there are other authors, they declare that they have no known competing financial interests or personal relationships that could have appeared to influence the work reported in this paper.

## Data Availability

All data generated or analysed during this study are included in this published article and its supplementary information files.

## Appendix A Supplementary data

Supplementary material Image 1
