# Supplementary material for: Ensuring vaccine cold chain integrity: A rapid and low-cost test for identifying heat-exposed sucrose-containing vaccines
Source: Int J Pharm X. 2025 Dec 11;11:100467. doi: 10.1016/j.ijpx.2025.100467 (PMC12774715; doi:10.1016/j.ijpx.2025.100467)
Supplement: Supplementary file 1 — Supplementary material [file mmc1.docx]

**Supplementary Material for Arman BY, *et al*. Int J Pharmaceutics: X. 2025**

Ensuring vaccine cold chain integrity: A rapid and low-cost test for identifying heat-exposed sucrose-containing vaccines

**Table S1.** The concentration of glucose in vaccines and its fold-change increase after 7-days exposure to elevated temperatures. These four vaccines showed a statistically significant increase in glucose at the elevated temperatures (Ordinary one-way ANOVA with Dunnett’s multiple comparisons tests p<0.05).

| **Vaccine** | **Glucose concentration* (μM)** | | | **Fold-change** | |
| --- | --- | --- | --- | --- | --- |
|  | **2-8 °C** | **37 °C** | **45 °C** | **37 vs 2-8 °C** | **45 vs 2-8 °C** |
| Bexsero | 5.63 | 25.49 | 51.59 | 4.5 | 9.2 |
| COMIRNATY | 2.34 | 22.13 | 64.08 | 9.6 | 27.6 |
| COVISHIELD | 26.21 | 73.46 | 164.85 | 2.8 | 6.3 |
| Nimenrix | 0.43 | 0.47 | 0.51 | 1.1 | 1.2 |

*Mean concentration of N=2

**Table S2**. Concentrations of seven analytes measured in vaccine samples using a biochemical analyser after exposure to different altered temperature conditions. Values in bold indicate if the analytes could be detected and quantified showing their mean concentrations (± standard deviation) of eight measurements. All other values with the less than symbol (<) were below the limit of quantitation and the lower limit of quantitation is shown

| **Condition** | **Calcium (mM)** | **Chloride (mM)** | **Magnesium (mM)** | **Phosphate (mM)** | **Potassium (mM)** | **Protein (mg/L)** | **Sodium (mM)** |
| --- | --- | --- | --- | --- | --- | --- | --- |
| Bexsero^*^ | | | | | | | |
| 4 °C | < 0.50 | **110.92 ± 1.09** | < 0.74 | < 1.50 | **2.52 ± 0.04** | < 68 | **107.30 ± 1.25** |
| 3× FT | < 0.50 | **110.10 ± 0.81** | < 0.74 | < 1.50 | **2.52 ± 0.04** | < 68 | **106.93 ± 0.73** |
| RT | < 0.50 | **110.17 ± 0.82** | < 0.74 | < 1.50 | **2.52 ± 0.04** | < 68 | **106.73 ± 0.92** |
| 37 °C | < 0.50 | **110.70 ± 0.95** | < 0.74 | < 1.50 | **2.52 ± 0.04** | < 68 | **107.18 ± 0.94** |
| 45 °C | < 0.50 | **110.95 ± 1.01** | < 0.74 | < 1.50 | **2.57 ± 0.07** | < 68 | **107.68 ± 1.21** |
| COMIRNATY | | | | | | | |
| 4 °C | < 0.50 | < 20.0 | < 0.74 | < 1.50 | < 1.0 | **184.88 ± 13.41** | < 20.0 |
| 3× FT | < 0.50 | < 20.0 | < 0.74 | < 1.50 | < 1.0 | **170.63 ± 7.19** | < 20.0 |
| RT | < 0.50 | < 20.0 | < 0.74 | < 1.50 | < 1.0 | **186.63 ± 5.24** | < 20.0 |
| 37 °C | < 0.50 | < 20.0 | < 0.74 | < 1.50 | < 1.0 | **190.88 ± 9.91** | < 20.0 |
| 45 °C | < 0.50 | < 20.0 | < 0.74 | < 1.50 | < 1.0 | **194.50 ± 10.24** | < 20.0 |
| COVISHIELD | | | | | | | |
| 4 °C | < 0.50 | **39.26 ± 0.26** | **1.04 ± 0.02** | < 1.50 | < 1.0 | < 68 | **34.84 ± 0.67** |
| 3× FT | < 0.50 | **39.25 ± 0.29** | **1.04 ± 0.04** | < 1.50 | < 1.0 | < 68 | **34.89 ± 1.12** |
| RT | < 0.50 | **39.28 ± 0.24** | **1.03 ± 0.03** | < 1.50 | < 1.0 | < 68 | **35.31 ± 1.39** |
| 37 °C | < 0.50 | **39.44 ± 0.27** | **1.04 ± 0.01** | < 1.50 | < 1.0 | < 68 | **35.21 ± 1.21** |
| 45 °C | < 0.50 | **39.33 ± 0.26** | **1.03 ± 0.03** | < 1.50 | < 1.0 | < 68 | **35.18 ± 0.65** |

^*^Calculation based on six measurements due to the limited sample; 3× FT, three cycles of freeze-thaw; RT, room temperature (20 ± 1 °C).


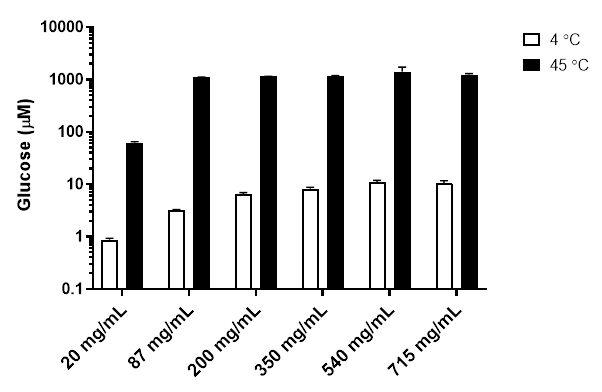


**Figure S1.** Sucrose samples were made up in water at various concentrations from 20 to 715 mg/ml and stored at both 4 °C and 45 °C for 7 days. Glucose concentrations were then measured using the bioluminescence assay. Error bars show the standard deviations from two measurements.
